# Progress of Epidemics in Forty Towns and Districts

**Published:** 1857-07

**Authors:** 


### PROGRESS OF EPIDEMICS.

### LOCAL REPORTS OF EPIDEMIC AND ENDEMIC DISEASES

*During the Months of March, April, and May,* 1857.

| Place. | County. | Lat. | Long. | Observer. |
|---|---|---|---|---|
| St.Mary, Scilly | Cornwall - | 49.50 N. | 6.24 W. | J. G. Moyle, Esq. |
| Teignmouth - | Devonshire - | 50.32 N. | 3.29 W. | W. C. Lake, Esq. |
| Odiham - - | Hampshire - | 51. 8 N. | 1. 3 W. | J. McIntyre, M.D. |
| Canterbury - | Kent - - | 51.17 N. | 1. 4 E. | G. Rigden, Esq.<br>W. Haffenden, Esq. |
| Chatham - | Kent - - | 51.21 N. | 0.14 E. | F. J. Brown, M.D. |
| Wandsworth - | Surrey - - | 51.28 N. | 0. 7 W. | G. E. Nicholas, Esq. |
| Putney - - | Surrey - - | 51.28 N. | 0. 8 W. | R. H. Whiteman, Esq. |
| Up. Holloway | Middlesex - | 51.32 N. | 0.03 E. | W. B. Kesteven, Esq. |
| Swansea - | Glamorgansh. | 51.38 N. | 3.50 W. | W. H. Michael, Esq. |
| Aspley Guise - | Bedfordshire - | 52. 1 N. | 0.37 W. | J. Williams, M.D. |
| Saffron Walden | Essex - - | 52. 3 N. | 0.12 E. | T. Spurgin, Esq.<br>H. Stear, Esq. |
| Bedford - - | Bedfordshire - | 52. 8 N. | 0.51 W. | T. H. Barker, M.D. |
| Newpt.Pagnell | Buckinghams. | 52.10 N. | 0.42 W. | G. O. Rogers, Esq. |
| Sharnbrook - | Bedfordshire - | 52.12 N. | 0.40 W. | R. S. Stedman, Esq. |
| Wellingbro' - | Northamptons. | 52.20 N. | 0.40 W. | B. Dulley, Esq. |
| Beccles - - | Suffolk - - | 52.25 N. | 1.48 E. | W. E. Crowfoot, Esq. |
| Thetford - | Norfolk - - | 52.26 N. | 0.45 E. | H. W. Bailey, Esq. |
| Stourbridge - | Worcestersh. - | 52.28 N. | 2.32 W. | A. Freer, Esq. |
| Dudley - - | Staffordshire - | 52.30 N. | 2.10 W. | J. H. Houghton, Esq. |
| Barrowden - | Rutland - | 52.34 N. | 0.38 W. | H. J. Swann, Esq. |
| Wisbeach - | Cambridgesh. | 52.39 N. | 0. 5 E. | W. H. Hole, Esq. |
| East Dereham | Norfolk - - | 52.40 N. | 0.57 E. | J. Vincent, M.D. |
| Pontesbury - | Shropshire - | 52.43 N. | 2.50 W. | Wm. Eddowes, Esq. |
| Nottingham - | Nottinghamsh. | 52.50 N. | 1.10 W. | T. Robertson, M.D. |
| Burton-on-Trt. | Staffordshire - | 52.53 N. | 1.53 W. | S. Thomson, M.D. |
| Oswestry - | Shropshire - | 52.53 N. | 2.59 W. | P. Cartwright, Esq. |
| Wrexham - | Denbighshire - | 53. 2 N. | 3. 1 W. | E. Williams, M.D. |
| Hawarden - | Flintshire - | 53.11 N. | 3. 2 W. | T. Moffat, M.D. |
| Lincoln - | Lincolnshire - | 53.12 N. | 0. 5 W. | S. Lowe, Esq. |
| Alford - - | Lincolnshire - | 53.15 N. | 0. 6 E. | R. U. West, M.D. |
| Gainsborough | Lincolnshire - | 53.23 N. | 0.47 W. | D. Mackinder, M.D. |
| Liverpool - | Lancashire - | 53.24 N. | 2.59 W. | Thos. Bickerton, Esq. |
| Warrington - | Lancashire - | 53.25 N. | 2.32 W. | C. N. Spinks, Esq. |
| Wigan - - | Lancashire - | 53.32 N. | 2.33 W. | Jephtha Hussey, Esq. |
| Bolton - - | Lancashire - | 53.35 N. | 2.19 W. | W.H.Pendlebury,Esq. |
| Bramley - | Yorkshire - | 53.49 N. | 1.38 W. | J. N. Radcliffe, Esq. |
| York - - | Yorkshire - | 53.58 N. | 1. 3 W. | W. Procter, Esq. |
| Wst. Auckland | Durham - | 54.45 N. | 1.40 W. | G. Todd, Esq. |
| Rothbury - | Northumberld. | 55.25 N. | 1.50 W. | E. C. Summers, Esq. |
| Lerwick - | Shetland Isles | 60. 9 N. | 1. 8 W. | G. W. Spence, M.D. |

### QUARTERLY STATEMENT—No. X.

[*The dates denote that the disease appeared in the weeks then ending.*]

SCARLET FEVER.

Canterbury..Mar. 13-20, April 10-17, May 8-22
Swansea..All April, May 8-15,
Bedford..April 10-17
Beccles..March 20-27
Thetford..April 10, 24
Dudley..March 20, all April
Barrowden..May 1
Pontesbury..All March
Burton-on-Trent..May 15, 29
Wrexham..Mar. 20-27, all April & May

Liverpool..Every week [1-15 29
Warrington..All Mar., Apl. 3 17, May
Bolton-le-Moors..March 6-20 April 17-24, May 1-15
York..March 13-20, April 10
West Auckland..All March, April 3
Rothbury..May 15

MEASLES.

Chatham..March 27
Wandsworth..April 24
Upper Holloway..March 20, May 8-15
Saffron Walden..Mar. 13-27, all April
Bedford..All Mar., April 3 [& May
Newport Pagnell..May 1
Sharnbrook..All March, April 3-10
Stourbridge..May 15
Dudley..Mar. 6, 20, Apl. 10-24, May 1,
Wisbeach..March 6-13 [15-29
Pontesbury..Every week
Nottingham..April 10
Hawarden..April 24, May 8-15, 29
Lincoln..Mar. 27, April 3-17, May 8-29
Alford..March 6, May 1
Gainsborough..Every week
Liverpool..Mar. 6, 20-27, May 1, 15, 29
Warrington..April 10-24, all May
Bolton-le-Moors..March 20-27, April 10-24, all May
York..Mar. 13-20, April 3-17, all May
Rothbury..April 10-24

SMALL-POX.

Canterbury..April 17-24
Chatham..March 20
Swansea..May 22-29
Sharnbrook..April 3-10
Stourbridge..Ma. 6, 20, April 17, May 29
Dudley..Mar. 6, 27, April 3-10, all May
East Dereham..All May
Nottingham..May 29
Burton-on-Trent..May 29
Liverpool..Every week except Mar. 13
Bolton-le-Moors..Mar. 27, April 3-17,
York..April 3-17, May 8-22 [May 8-22
West Auckland..All March
Lerwick..April 3

HOOPING COUGH.

Canterbury..All Mar., Apl. 3-17, May 1-
Chatham..March 6, April 10-17 [22
Wandsworth..April 24, May 1
Putney..April 17 [May 1-8
Upper Holloway..Mar. 6-20, Apl. 10 24,
Swansea..All March and April, May 1-8, 22
Saffron Walden..All Mar., April 3-10
Bedford..All March & April May 1-8
Newport Pagnell..Every week
Sharnbrook..Every week
Wellingborough..All Mar., April 3-10
Beccles..March 6-13, 27, April 3
Stourbridge..March 27, April 24
Dudley..Mar. 13, 27, Apl. 3, 17, May 1,
Barrowden..Every week [15-22
Wisbeach..All May
Pontesbury..March 6-20, May 8-29
Nottingham..Mar. 6, April 10, May 15
Oswestry..Every week
Wrexham..April 24, all May
Lincoln..March 6-13
Gainsborough..May 29
Liverpool..Every week
Warrington..Every week
Bolton-le-Moors..All Mar., April 3-10,
Bramley..Every week [May 1-15
York..March 13-20

CROUP.

Canterbury..April 10-17, May 8-15
Chatham..April 3, 17
Upper Holloway..Mar. 27, April 10-17
Swansea..March 20-27, April 3
Saffron Walden..May 1, 22
Bedford..March 27
Newport Pagnell..April 10, 24, May
Beccles..May 1 [15-22
Thetford..April 17, May 8
Stourbridge..May 1
Pontesbury..March 13-20
Nottingham..April 3, May 15-22
Burton on-Trent..March 27, May 29
Oswestry..All May
Wrexham..March 13-20, April 10-24
Liverpool..Mar. 13, 27, all Apl., May 1-8
Warrington..Mar. 20, Apl. 3-10, May 8-
Bolton-le-Moors..April 3-10 [15, 29

CATARRH.

St. Mary's, Scilly..April 24, May 1-15
Teignmouth..March 6, 20, April 10, 24, May 1, 15, 29
Odiham..All Mar., April 3, 24, May 1,
Canterbury..April 3 [15, 29
Chatham..Every week except May 29
Wandsworth..March 13, April 3, May 15-22
Putney..All Mar., April 3-10, May 1-8
Upper Holloway..All Mar., May 1-22
Swansea..Mar. 13-27, April 3-10, May
Aspley Guise..March 13-27 [8-22
Saffron Walden..Every week
Newport Pagnell..All March & April, May 1, 22-29
Sharnbrook..All Mar. & April, May 1-15
Wellingborough..All March & April
Beccles..Mar. 6-20, all April, May 22-29
Thetford..All Mar., April 10-17, May
Stourbridge..April 3 [8-22
Dudley..March 20, April 3
Wisbeach..March 6-13, all May
East Dereham..April 17-24, May 1-8
Pontesbury..March 20-27
Nottingham..Mar. 6, 20-27, all April, May 1-22

Burton-on-Trent..All Mar.&Apl.,May
Oswestry..April 10-24, all May [1-8
Alford..April 24
Gainsborough..Every week
Liverpool..Every week
Bolton-le-Moors..Mar. 6-20, all April
Bramley..March 6-13
Rothbury..Every week
Lerwick..Mar. 6-13, 27, April 3-10, 24, May 1-8, 22-29

INFLUENZA.

St. Mary's, Scilly..All Mar., Apl. 3-17
Teignmouth..Mar.6-13, all April, May
Odiham..All March [1-15
Chatham..Every week
Wandsworth..March 6, 27, April 3
Upper Holloway..April 10-17
Saffron Walden..Every week
Bedford..March 20-27, April 3
Newport Pagnell..Every week
Sharnbrook..All April and May
Wellingborough..All March, April 3
Beccles..AllMarch, April 10, 24, May 22 29
Thetford..March 6-23, 27, all April, May 1, 15-22
Lincoln..All Mar., April 24, May 1-8
Alford..March 6, April 3-10
Liverpool..Every week
Bolton-le-Moors..Mar. 13-27, Apl. 10-
York..April 10-24, May 8 [17
Lerwick..All Mar., April 10-24, May 1

ERYSIPELAS.

Odiham..March 6, May 1-8
Canterbury..March 6, April 3, May 29
Chatham..March 6, 27
Wandsworth. Mar. 6, 20-27, April 24,
Putney..April 10, 24 [May 15
Upper Holloway..March 13-20
Swansea..April 17, May 22-29
Saffron Walden..Mar. 6, Apl.3, May 29
Newport Pagnell..March 20
Beccles..May 8
Thetford..March 6, April 10, May 8
Stourbridge..May 15
Dudley..April 3
Wisbeach..May 8-29
Pontesbury..March 6-13, May 22-29
Nottingham..May 1
Burton-on Trent..May 22
Wrexham..March 27, April 3-17
Lincoln..May 25-29
Alford..April 3, May 1
Gainsborough..Mar. 6, 20, April 8-15
Liverpool..March 13, 27, April 3, 24, May 1-8, 22
Warrington..Mar.6, Apl.10-17, May29
Bolton-le-Moors..Mar.27, April 3, May
Bramley..March 6 [1-15
Rothbury..May 15

CHOLERA (ENGLISH).

Chatham..March 27, April 3
Hawarden..May 1
Liverpool..March 6, May 29

AGUE.

Canterbury..Every week
Chatham..Every week
Wandsworth..April 24
Putney..May 8
Aspley Guise..May 29
Saffron Walden..April 3, 17-24, May 1
Bedford..April 10 24, all May
Newport Pagnell..April 24, May 1-8
Sharnbrook..Mar. 20-27, April 10-17, May 29
Wellingborough .April 24, all May
Thetford..April 10, May 1, 22-29
Barrowden..April 3, 17, May 1, 22
Wisbeach..Every week
East Dereham..April 17-24, all May
Lincoln..March 13-27, April 4
Alford..May 8-15
Gainsborough..May 29
Warrington..March 27

REMITTENT FEVER.

Teignmouth..Mar. 13-27, April 3-10, 24, May 1
Odiham..March 6-13, May 1-15
Canterbury..Every week
Chatham..May 8-22
Upper Holloway..May 1-8
Swansea..Mar. 20-27, April 3-10, May
Saffron Walden..May 15-22 [8-22
Beccles..March 13-20, April 3-17
Thetford..March 6-13, May 8-15, 29
Stourbridge..April 17
Wisbeach..April 24, all May
Nottingham..May 15-22
Oswestry..All March, April 3-10
Lincoln..April 24, May 1-8
Alford..March 27, all April
Wigan..All April and May
Bolton-le Moors..Mar.23 20, all April,
York..Mar. 13-20, April 10-24 [May 8

DIARRHŒA.

Teignmouth..March 13, April 3-10, 24, May 1-8, 22-29
Odiham..Mar.13, April 10, 24, May 22
Canterbury..May 29
Chatham..All Mar., April 17-24, May 1
Wandsworth..Mar.27, all Apl. May8 29
Swansea..Mar. 6, April 3-17, May 1, 15
Aspley Guise..March 27, April 3-10, May 1 [May 8-29
Saffron Walden..All Mar., April 3-10,
Bedford..March 6, 27, April 3-10, 24, May 1, 29 [and May
Newport Pagnell..Mar. 13, 27, all April
Sharnbrook..Mar.20-27, all Apl.&May
Wellingborough..April 10-24, all May

Beccles..April 17, May 22-29
Thetford..All Mar.,April 3,24,May1-8,
Stourbridge..Mar. 27, April 24 [22-29
Dudley..Every week except April 10
Barrowden..April 17, May 22
Wisbeach. May 8-15
Nottingham..March 6-23, May 1, 29
Burton-on-Trent..March 27
Wrexham..March 6-13
Hawarden..March 6-13, 27, April 17-24, May 8-15, 29
Alford..March 6, 20, April 3 17
Gainsborough..Every week
Liverpool..Every week
Warrington..March 8-29
Wigan..May 15-29
Bolton-le-Moors..Mar.27 April 3 May [1-15
Rothbury..May 1-15
Lerwick..May 8, 22

DYSENTERY.

Teignmouth March 13, 27, May 22
Chatham..March 6
Wandsworth..Mar.27,April3 10,May1
Putney..April 17, May 1-8
Bedford..April 3 10
Newport Pagnell..April 3-17
Sharnbrook..March 27
Beccles..March 13
Wisbeach..March 6-13
Pontesbury..March 27
Nottingham..March 20 [29
Wrexham..Mar.6-13,April 10, May15-
Gainsborough..All March, April 24, all May [May 1-8
Liverpool..March 13, 27, April 3, 24,
Warrington May 15-29
Wigan..April 24
Bolton-le Moors..May 8-22
Bramley..Mar. 6, April 17-24, May 15
York..May 8, 22

TYPHUS.

Teignmouth..March 20, April 3-10
Odiham..March 13
Canterbury..Every week
Chatham..Every week
Wandsworth..April 3, May 1, 29
Bedford..April 10-17
Sharnbrook..March 20
Beccles..May 8-15
Thetford..March 6,27,April 17,May 1
Dudley..Every week except March 6
Wisbeach..March 6-13
Pontesbury..April 10-24, May 1-15
Nottingham..Mar.20-27,Apl.10, May
Oswestry..April 17-24, all May [15
Gainsborough..May 8-29
Liverpool..Every week
Warrington..May 29
Wigan..Map 8-29
Bolton-le-Moors..Mar. 6-13, 27, April 3 10, May 1-8, 22-29
Rothbury..Every week
Lerwick..March 6-20, April 17-24

PUERPERAL FEVER.

Putney..May 1
Saffron Walden..March 27, April 3
Newport Pagnell..May 8
Alford..March 20
Rothbury..May 8
Lerwick..April 17

CARBUNCLE.

St. Mary's, Scilly..May 1-8
Teignmouth..April 24
Canterbury..Every week
Chatham..March 20-27, April 3
Aspley Guise..April 17-24, May 1
Saffron Waldon..March 6-13, May8,22
Newport Pagnell..May 8-29
Beccles..March 20, May 8-15
Thetford..March 13, April 10, 24
Stourbridge..May 15-22
Dudley..May 22
Wrexham..April 3-10
Liverpool..Mar. 20, Apl. 3, May 1-8, 29
Bolton-le-Moors..April 10, May 1-8
Bramley..March 6
York..Mar.13-20,Apl.3,17,May1,15,29
Lerwick..April 17

VARICELLA.

Wandsworth..March 6

ACUTE RHEUMATISM.

Wrexham..All March, April 3-10

---

### ADDITIONAL OBSERVATIONS.

*St. Mary's, Scilly.*—Mr. Moyle states that the quarter was very healthy.

*Canterbury.*—Mr. Rigden writes: "During the past quarter there was less than the usual amount of sickness observed at this period of the year. Most of the cases of scarlet fever assumed a mild character. Hooping cough continued to prevail throughout the winter months; but with the return of warmer weather the children with this complaint did not require medical attendance. More than the usual number of patients were afflicted with intermittent and remittent fevers, also with intermittent neuralgia; but these were generally much relieved by the administration of sulphate of quinine. Continued fevers still prevailed, but were mostly limited to the lower parts of the city, where the poorer classes reside. There was but little if any abatement in the number of patients afflicted with furunculoid diseases. During the month of March there were deaths registered as resulting from scarlet fever, typhus, hooping cough, and catarrh; during April from scarlet fever, croup, and small-pox; during May from scarlet fever, fever, and diarrhœa."

Mr. Haffenden has forwarded the following quarterly returns, which has been drawn up by the Epidemiological Committee of the East Kent and Canterbury Medical Society.

"*Abstract of Meteorological Observations for the Spring Quarter*, 1857.

| | *March.* Deg. | *April.* Deg. | *May.* Deg. |
|---|---|---|---|
| Highest temperature in the day time .... | 50 | 55 | 66 |
| Lowest .............................. | 32 | 38 | 44 |
| Mean ................................ | 40·90 | 46·43 | 55·22 |
| Highest temperature at night .......... | 49 | 50 | 56 |
| Lowest .............................. | 26 | 32 | 37 |
| Mean ................................ | 36 61 | 38·93 | 46·58 |
| Highest reading of barometer .......... | 30·36 | 30·14 | 30·11 |
| Lowest .............................. | 29·12 | 28·85 | 29·44 |
| Mean ................................ | 29·758 | 29·465 | 29·836 |
| Amount of rain in inches .............. | 1·10 | 2·11 | 1·23 |

Direction of wind (the number indicates the number of days certain winds prevailed). *March*—NE. 1, E. 4, SE. 5, S. 4, SW. 6, W. 6, NW. 5. *April*—N. 5, NE. 2, E. 2, SE. 2, S. 6, SW. 6, W. 6, NW. 1. *May*—N. 5, NE. 10, E. 3, SE. 2, S. 2, SW. 7, W. 2.

*Abstract of the returns of Zymotic diseases during the Spring Quarter*, 1857.

"The number of cases of zymotic disease, reported during the spring quarter, was 104; being a great decrease in the number of the same quarter last year. Five cases have proved fatal; two from scarlatina; two from continued fever; and one from typhus. Of scarlatina, twenty-two cases were returned; this shows a great decrease on the previous quarter. Two of the number died. One of these was a female twenty-three years of age, who was a patient in the hospital, and had been under treatment there a month, with strumous disease of the elbow-joint. The eruption was very vivid, but the throat symptoms were not severe; she died from exhaustion four days after the commencement of the attack. There had not been any scarlatina in the hospital for a considerable period prior to this case; nor has there been any since. This case occurred in April. The other fatal case from scarlatina was a female eight years of age, who suffered from malignant tonsillitis, had a rigor on the eleventh day, followed in a few hours by anasarca, and died on April 18th, from apnœa and exhaustion. Under the head of fever twenty-two cases were recorded, though not all of the same class. The larger proportion was continued ; only one of the number was typhus, and that proved fatal, in a female fourteen years of age. The disease was complicated with pneumonia. Two deaths occurred from continued fever ; both in young female children. Ague was more prevalent in Canterbury and the neighbourhood in the last quarter, than for very many months past. Thirty cases were reported, all of which recovered. The tertian type was by far the most common, and next to it the quotidian ; only one or two of the quartan were noticed. Quinsy was noticed by one or two observers as being rather more prevalent than usual at this time of year ; seven cases came under notice, which all recovered. Of diarrhœa, five cases only were returned ; these occurred in the month of April, and were mild in character. Erythema and erysipelas appeared in a few instances ; not quite so many cases were returned as in the last quarter."

*Chatham.*—Dr. Brown writes : "The wind was easterly during fifty-one days of March, April, and May. Cases of influenza were very numerous, but less fatal to the aged than usual. Diarrhœa prevailed ; and there were a few cases of Spring cholera. Typhoid fever, attended by great adynamia, and succeeded by pyæmic abscesses in the neck, and by dropsy, was prevalent in April and May. The localities that suffered most were the low lying parts of Rochester, which yielded the bulk of the cholera cases in former years. Some cases occurred on St. Margaret's Hill, but they were confined to a few houses, in a very defective hygienic condition, tenanted by poor people. Typhoid fever in Rochester and Chatham usually turns on the fourteenth or seventeenth day ; but in the present epidemic, although some cases turn early, many go on to the twenty-eighth day. One case terminated on the twenty-first day by laryngitis. I have not seen a single case of typhus during the last three months. Remittent fever appeared in May, amongst children. This is a rare fever in Rochester and Chatham. The cases were characterised by head symptoms ; viz., drowsiness, flushed face, startings, delirium, vomiting, costiveness, frequent pulse, strawberry tongue, and dilated pupils. The throat has been affected by diphtheritis. There have been paroxysms of vomiting, with aggravation of head symptoms, followed by remissions. One case died from convulsions,—probably by the supervention of cephalitis. I mistook the nature of the case, and registered it as acute hydrocephalus —fifteen days. But it began with catarrh and hoarseness, and epistaxis occurred during the course of the complaint. Another case died with symptoms of inflammation of the liver. This case was also mistaken. It was registered as diphtheria—hepatitis. The treatment that speedily cures these cases, is free leeching of the head, with purgatives. No quinine has been used. Intermittent fever prevailed in its masked form to a greater extent than I have ever known it to do in this place. One case of well marked quartan fever was attended by alarming symptoms,—blue collapse, with quasi-mania. It readily yielded to quinine in large doses. The incomplete form of ague was the most prevalent. The symptoms were cold sweats, sense of constriction round the head, eye-brow ague, face-ache, arm-ache, malarious sciatica, etc. The last case of melæna occurred last quarter (end of February)."

*Wandsworth.*—Mr. Nicholas reports disease of the zymotic class to have been trifling, both in amount and intensity, during the past quarter.

*Putney.*—Mr. Whiteman says: "Not a single death resulting from zymotic disease was registered in this sub-district during the quarter. Diarrhœa prevailed at the early part of May, two or three somewhat severe cases coming under treatment about that time. Catarrhal affections were noticed in nearly every week. There occurred in my practice, during the thirteen weeks, two cases of erysipelas, one of hooping cough, and one of puerperal fever. Of small pox, scarlatina, or measles, I had not a single case during the quarter; nor have I heard of the occurrence of any cases in this parish. The case of ague in May was imported from Sheerness. There was a most manifest improvement in the public health of this parish during the quarter; and I anticipate a still greater one when the drainage operations, now in progress, are complete. The deaths from zymotic diseases, during the year 1856, were thirteen in number; and, with scarcely an exception, they took place in localities entirely undrained, where cesspools abounded, and where consequently the well water was of the worst description."

*Swansea.*—Mr. Michael writes: "The past quarter, like the preceding one, was marked by great comparative immunity from disease. Several cases of sudden death, however, occurred. Hooping cough gradually declined in severity and extent, and scarlet fever too almost, if not entirely, disappeared. A case of this disease, however, occurred on May 8th, proving fatal from blood-poisoning in thirty hours; the other children attacked, two in number, had the disease in a mild form. The house was in an elevated situation on the east side of a hill, facing the sea. Two cases of small pox occurred, both imported; one was a German girl, recently arrived from Cardiff, where the disease was epidemic; the other, from Briton Ferry, in the neighbourhood of Neath, where cases had also occurred. Both were cases modified by vaccination, and terminated favourably. No other cases have occurred."

*Aspley Guise.*—Dr. Williams states that the stormy and changeable weather of the past spring produced a prevalence of pulmonary affections in animals, and in the human subject. In the former some cases were fatal.

*Saffron Walden.*—Mr. Spurgin writes: "Vegetation was somewhat backward at first from the coldness of the atmosphere in the earlier part of the quarter; but it is now proceeding very favourably, with a promise of good crops of fruit and corn. Animals were generally healthy, from the dryness of the season. Measles were very prevalent in this district, generally of a mild character. Few fatal cases occurred; none in my own private practice."

Mr. Stear says that measles extensively affected both children and adults. In many instances bronchitis followed, and was fatal in several cases. Vegetation was very healthy.

*Thetford.*—Mr. Bailey sends the following observations: "March, 1857. The weather during this month was very variable, and the fluctuations of the barometer were sudden; the mean of atmospheric pressure for the month was 29·80°, and the range 14·10°. The amount of rain was under half an inch. The mean temperature of the month was 47°, and the range, from the highest to the lowest, 38°. From this variation of climate we had under notice several cases of catarrh and influenza, which may be considered epidemic. The latter, in some elderly people, proved fatal. With children exposed to the cold winds, and in very young infants, many cases terminated fatally in a few days. Two cases of remittent fever occurred in labouring men, who, from great poverty and exposure to the wet and cold, became affected with the disease, which continued some days. Occasionally a relapse took place; but as the temperature increased, they ultimately entirely recovered. Erysipelas occasionally made its appearance, even in those who were never subject to it before, producing much febrile action, and lasting many days, leaving the skin exceedingly sensitive to the cold airs, etc. Diarrhœa was very prevalent throughout the month, both in children and in adults, and also in women during their month of confinement. The great exhaustion occasioned by it required a considerable time to recover, and in some instances relapses took place. It was more epidemic than I have known, at this season, for many years. Pleurodynia also prevailed among the working classes, attended sometimes with cough, but more generally without. So severe were some cases, that perfect inability to work ensued, and the cases required active treatment. Many cases of rheumatism, tic douloureux, sciatica, cynanche tonsillaris, and two cases of continued fever, occurred during the month, probably from the influence of the weather. The casual diseases of the month were hæmoptysis, abortion, hydrocele, hysteria, abdominal dropsy, spasms, dyspepsia, uterine cancer, and some cutaneous eruptions. In this locality, a great mortality existed amongst the flocks: many farmers having lost their ewes and lambs from the murrain, which, by the shepherds, is attributed to the artificial manures, rendering the turnips too rank and less nutritious. May not this state be caused by a certain effluvium, or miasma, emanating from the soil which has been highly manured in this way? Pneumonia, sore throats, and febrile affections, were prevalent this month among the horses and cattle.

"April. The temperature of this month gave, comparatively, two summers and two winters. During the first fortnight it was warm, the thermometer rising to 65°, and then falling on the 12th to 50°, and in the evening to 37°, and on the grass to 30°. For four or five days it continued very cold, and severe frosts occurred at night. Afterwards summer commenced again; the temperature rose to 67° degrees on the 19th for a few days. But from the 23rd to the end, the month was cold, with hailstones, snow, and sleet; the night temperature was many degrees below the freezing point. These changes had much effect upon the human system, and produced many rheumatic cases, cynanche tonsillaris, and one fatal case of croup in a boy four years of age. Catarrh and influenza were also very prevalent. Two cases of scarlatina occurred in the neighbourhood; but these I believe to have been taken from a distant place where they had visited. It did not extend any further. Erysipelas occurred in one case only; it attacked the head and face, producing much constitutional derangement, but terminated favourably. Diarrhœa was not so prevalent this month; two cases only came under observation. Bronchitis and pneumonia, from the sudden changes, were more general. Continued fever occurred in one case only, in an elderly workman; it arose from great exposure to heat and cold, the man being a blacksmith by trade. Two cases of furunculus occurred during the month; one in a gentleman, who had eight at the same time, in various parts; they confined him entirely to his bed for many days, producing great constitutional irritation, and much fever. This complaint has been very prevalent of late, attacking the working classes, as well as others in the higher grade of life. It seemed to be the epidemic during the month. The casual diseases under treatment were apoplexy, menorrhagia, gastralgia, uterine cancer, gonorrhœa, gout, ophthalmia, and hæmorrhoids. Similar diseases as in last month still continued among the cattle, and especially sheep, but not to the same extent.

"May. During the early part of the month, the temperature did not reach higher than 55°, even in the middle of the day, but at night, and on the grass, it was as low as 28°; and for several nights we had frosts, which damaged the young potato plants. The following week the temperature increased; and on the 15th it rose as high as 75°, and at night, the same day, sank to 41°—a difference of 34°. These changes gave rise to pectoral complaints, which were very prevalent. Cases of catarrh, influenza, bronchitis, and pneumonia, were at the same time under medical treatment; they were not confined to the class who are more exposed and predisposed, but extended to the higher classes, running through families. One serious case of typhus occurred in a poor working lad, of a weak constitution, who had been much exposed to the weather, besides being badly clothed. The disease ran through all its grades, and at the end of five weeks left him in a most weakened state; he is slowly recovering. Influenza and sore throats, quinsy and rheumatic affections, in a few instances, came under treatment; also one case of enteritis, which suddenly attacked a poor man while at work. He was brought from the field home and required the most active treatment; he recovered. Only one case of erysipelas occurred during the month, which attacked the leg. The patient had been occasionally subject to the disease. By the application of a solution of nitrate of silver, he soon recovered. During this month several cases of remittent fever took place, apparently arising from atmospheric influence, as the subjects of attack were in a good position of life. The casual diseases in this month were phthisis, tic douloureux, hepatitis, lumbago, procidentia uteri, headache, and some cutaneous affections. The more favourable state of weather lessened the diseases amongst the cattle; the flocks are doing remarkably well, and healthy.

*Record of Deaths in Twenty-one Parishes* (*population* 9,574) *from the Registrar's Book.*

| *March.* | | *April.* | | *May.* | |
|---|---|---|---|---|---|
| Drowning ........ | 1 | Hooping cough .... | 1 | Bronchitis ........ | 4 |
| Hooping cough .... | 1 | Bronchitis ........ | 1 | Pulmonary hæmorr.. | 1 |
| Debility .......... | 3 | Convulsions ...... | 1 | Old age .......... | 3 |
| Inflamma. of bowels | 1 | Senile gangrene of foot | 1 | Atrophy .......... | 2 |
| Diarrhœa.......... | 2 | Chronic asthma .... | 1 | Pneumonia........ | 1 |
| Old age .......... | 2 | Pulmonary hæmorr.. | 1 | Debility .......... | 1 |
| Consumption ...... | 1 | Old age .......... | 4 | Fever ............ | 1 |
| Acute rheumatism.. | 1 | Dropsy............ | 1 | Convulsions ...... | 1 |
| Bronchitis ........ | 1 | Diarrhœa.......... | 1 | Dropsy............ | 1 |
| Jaundice .......... | 1 | Croup ............ | 1 | Consumption ...... | 1 |
| | | Consumption ...... | 1 | | |
| | 14 | | 14 | | 16 |

*Stourbridge.*—Mr. Freer says that the cases of small-pox in the neighbourhood were less numerous than in the last quarter. Many cases of chicken-pox, some beginning with severe pain in the back, and high fever, appeared in the localities where a few months ago small-pox was rife. The one case of cholera reported, was in a boy aged six years. The only probable cause was eating rhubarb pudding. Death took place in sixteen hours. Influenza prevailed much among horses, characterised by early and marked debility.

*Dudley.*—Mr. Houghton writes: "Small pox, which was so prevalent in Dudley in the quarter last reported, when the deaths were fifty, somewhat declined during this quarter, the deaths being reduced to twenty-eight. Its place, however, was taken by measles, which caused twenty-four deaths. Hooping-cough and croup must be added to the new causes of mortality this quarter; the former caused five, and the latter eight deaths. The deaths from scarlet fever were 2; from erysipelas, 3; from diarrhœa, 16; from fever, 10; and from convulsions, 39. Diarrhœa, fever, and convulsions, continue to afford a constant addition to the mortality tables of Dudley. The great majority of the convulsions take place in children, whose health is impaired by the vitiated atmosphere in which they live. In two cases, hooping cough and measles occurred together. One patient, suffering from an attack of lepra, was seized with modified small pox, which seemed to have the effect of arresting the cure of the lepra. The deaths registered in Dudley for the last thirteen weeks, amounted to 366; the average for eight spring quarters was 238; hence the mortality was in excess of the average by 128; 105 of these deaths arose from zymotic diseases; the proportion of zymotic deaths to the whole being as 1 to 3·485. In London, at the corresponding period, the deaths were 14,232; the zymotic deaths, 2,459; the proportion of the zymotic deaths to the whole being as 1 to 5·786. The average number of deaths for ten corresponding quarters was 14,380. Consequently the deaths in London for the time specified, were 148 below the average. Thus the mortality of Dudley was greatly in excess of the average, whilst the mortality of London was much below it; and the proportion of zymotic deaths in Dudley was nearly twice what it was in London. No better proof can be given of the sanitary state of Dudley, and of the necessity for active sanitary interference. The readers of the *Sanitary Review* will have no difficulty in recognising the causes of this sad state of affairs, for which nothing is yet being done."

*Barrowden.*—Mr. Swann says that hooping-cough was severe at North Luffenham during the quarter. In two cases it produced great emaciation, with dyspnœa; several of the cases were attended by extreme ulceration of the fauces: iodide of potassium, with a tonic, had a remarkably beneficial effect. One death only occurred out of a great number of cases. During April, particularly at its commencement, bronchitis prevailed, and quickly removed some old people. In the latter part of the month the wind changed to north-east, the weather being dry and bitterly cold; several cases of rheumatism then appeared. Of ague there were several cases; one only at Barrowden, the others at Fineshade. It has been in the latter place three years. The cause of its outbreak and continuance is not known; but circumstances seem to point to its connection with water supply.

*East Dereham.*—Dr. Vincent writes: "A few cases of small pox were after vaccination; the disease was mild. There were no very bad cases, even when, as was the case in many, there had been no vaccination. One patient, who had previously (according to the parent's statement) well marked small-pox, had the disease for the second time. The pustules were very numerous all over the body. The disease ran a favourable course."

*Pontesbury.*—Mr. Eddowes states that the cases of typhus occurred in one family only. It was brought from Staffordshire, the neighbouring county, by a young man, one of the sons.

*Nottingham.*—Dr. Robertson says: "The quarter was a healthy one. The principal diseases were catarrh, croup, diarrhœa (principally infantile), and typhus. The latter was fatal in six cases, all in the lower part of the town. In March the weather was fine until about the 21st, when snow fell, and was succeeded by cold, dull weather, until the end of the month. The vegetation, which thus received a considerable check, remained in a backward state during the greater part of April, which was cold, and marked by storms of unusual heaviness. In the commencement of May the weather continued unfavourable until the 13th, after which warm showery days continued to the end of the month. The fall of rain was small, being only 1·8 for the whole month. I mentioned in my last report, that pleuro-pneumonia had been prevalent amongst cattle in this district; and I would refer those who are interested in the diseases of animals, to an interesting communication made to the Royal Agricultural Society in May by Mr. Paget, M.P. for this town, in which he states the result of experiments performed in 1853 by inoculating with matter, obtained from diseased lungs, those cattle in his dairy-herd which were attacked with the disease. This plan, which was successful in 1853, had entirely failed recently in the practice of Mr. Pyatt, veterinary surgeon of this town; a very high per centage of deaths resulting from its application. Professor Simonds, of London, who was consulted on the subject, stated his opinion that an inflammation, produced by a seton, dressed with irritating ointment, would be as efficacious as inoculation, and this belief was also held by some well informed men, who had investigated the disease on the continent. Mr. Pyatt tried the seton in some of the hovels where the disease was most rife; and, finding the results encouraging, extended the practice to a considerable portion of the herd. Previous to the application, Mr. Paget lost from 90 to 100 head of stock: more than three animals per week. Since the use of the seton, ten days had elapsed without a loss. The trial has been too short to lead to any satisfactory conclusion; but the further use of this harmless experiment is desirable, that some trustworthy conclusions may be obtained. I subjoin the quarterly table, for which I am, as usual, indebted to the kindness of Mr. White:—

| | *March.* | *April.* | *May.* |
|---|---|---|---|
| Barometer: Maximum | 30·22 | 29·94 | 29·96 |
| Minimum | 28·72 | 28·63 | 29·22 |
| Mean | 29·65 | 29·11 | 29·67 |
| Thermometer: Maximum | 56·3 | 68·1 | 75·2 |
| Minimum | 28·3 | 30·3 | 34·6 |
| Mean | 44·7 | 46·9 | 55·1 |
| Ozone | 7 | 5 | 6 |
| Rain | 2·3 | 2·7 | 1·8 |
| Direction of wind | W.&N.E. | SW.&E. | NE.&E. |
| Number of deaths | 141 | 104 | 110 |
| Ditto in 1856 | 154 | 157 | 144 |

"It is curious that measles, which killed forty-three persons in the corresponding quarter of 1856, was not once fatal in this quarter."

*Lincoln.*—Mr. Lowe says that there were a greater number of cases of ague during this quarter than usual; remittent fever was very prevalent in the fen districts. In other respects the quarter was more than usually free from epidemic and endemic diseases.

*Alford.*—Dr. West writes :—" April 11th. There is here, at the present date, quite an epidemic of acute pneumonia among young children. I have for the last three or four weeks scarcely ever been without six or seven such cases on my visiting list. Now, the epidemic of measles is on the decline ; and pneumonia is a frequent concomitant or sequela of measles. The pneumonia, which is now prevailing, appears to attack children who do not take measles, or who are insusceptible of that infection. Is it the case with epidemics that when there is a certain amount of insusceptibility—or, which is the same thing, where the infection is wearing out, patients may catch some one symptom of the disease ? It is unquestionable with regard to scarlatina and hooping cough, that we have frequent cases of *common sore throat*, when the former is, or has been, epidemic, and of *obstinate spasmodic cough*, during hooping-cough epidemics, with persons who have previously had those diseases, or are otherwise insusceptible of them in their complete form. In my own person, I always catch a sore throat, when in frequent attendance on scarlatina patients."

*Gainsborough.*—Dr. Mackinder writes :—" Of measles, which commenced at the end of the preceding quarter, we have had an epidemic, and the disease appears only to be abating now in consequence of there being but few houses whose juvenile inmates have escaped the infection. It assumed a mild type, a small minority of the cases only having been accompanied by pulmonic, ophthalmic, enteric, or renal complication. The mortality was insignificant, only two or three deaths having come to my knowledge. Stomatitis, in its various forms, was more than usually prevalent; and dysentery was common among children. A few cases of varicella were observed; but the small pox ceased with the cases reported in my last. I have seen but one person with mumps, one with hooping-cough, and one with gastro-enteric fever, (imported) ; but diarrhœa and catarrh were scarcely absent during the quarter. I had two or three cases of erysipelas in March and May ; a case of puerperal peritonitis in April ; and when the hot weather set in, a few of my parturient patients suffered more than common from *post partum* faintings and pains. Rheumatism had a few victims ; and a boy with tertian ague came under my notice on May 29th. He had been working for a couple of months in a wood, but was not aware that he had been exposed to the malaria of decomposing matter. Our mortality was small, and chiefly among the very old and the very young."

The subjoined Meteorological Report is furnished by Mr. Dyson:—"The weather, during the quarter has been remarkably seasonable and genial. The heat was generally in excess, and on the 27th of May, the maximum thermometer indicated 80). During the night of March 4th, it fell to 28); but generally it was slightly above the average. The development of ozone throughout the quarter, was remarkable, as compared with previous observations in this locality: few days have passed in which the test-paper has not been deeply discoloured. The amount of rain in March, was 1·76; in April, 2·63; and in May, 1·76; making a total of 5½ inches; being above the average of the same quarter as compared with previous years."

*Liverpool.*—Mr. Bickerton has sent the following weekly returns.

"March 6, 1857. During the week there were 221 deaths registered, being 24 below the average. Scarlatina caused 9 deaths; measles, 4; small-pox, 5; hooping-cough, 10; typhus, 4; diarrhœa, 2; convulsions, 11; atrophy, 15; scirrhus, 3; dropsy, 4; heart-disease, 11; diseases of the lungs, 89.

"March 14. There were registered in the week, 193 deaths, being 49 below the average of nine years. The causes were: scarlatina, 10; hooping-cough, 4; croup, 5; diarrhœa, 6; (5 infants); typhus, 1; disease of lungs, 77; disease of heart, 6; disease of brain and apoplexy, 16; convulsions, 16; delirium tremens, 1; dropsy, 9; erysipelas, 1; decay of nature, 24.

"March 21. During the week there were 222 deaths, being 25 below the corrected average. Scarlatina caused 10 deaths; small-pox, 2; hooping-cough, 1; fever, 5; diarrhœa, 4; diseases of lungs, 90; diseases of heart, 5; diseases of brain, 15; convulsions, 14; paralysis, 6; dropsy, 5; decay, 26.

"March 28. The deaths registered during the week were 236, being 13 less than the average. Scarlatina caused 6 deaths; measles, 1; small pox, 3 (2 not vaccinated); hooping-cough, 6; croup, 5; typhus, 1; continued fever, 5; diarrhœa, 0; diseases of lungs, 103 (very unusual); diseases of heart, 8; diseases of brain, 22; convulsions, 16; paralysis, 5; dropsy, 8; debility and natural decay, 32.

"April 4. 235 deaths were registered during the week, being about the average for this period. Scarlatina caused 7 deaths; measles, 0; small-pox, 4; hooping-cough, 5; croup, 7; typhus, 2; diarrhœa, 2; diseases of lungs, 82; diseases of heart, 9; diseases of brain, 20; convulsions, 19; dropsy, 8; erysipelas, 3; intoxication, 2; old age and debility, 23.

"April 11. During the week, 204 deaths were registered, being 22 less than the average. Scarlatina caused 5 deaths; measles, 0; small-pox, 0; hooping-cough, 5; typhus, 4; simple fever, 6; croup, 2; diarrhœa, 1; diseases of lungs, 85; diseases of heart, 7; diseases of brain, 15; convulsions, 21; paralysis, 3; diseases of liver, 2; dropsy, 3; debility and old age, 27.

"April 18. 195 deaths were registered, being 31 less than the average. Scarlatina caused 5 deaths; small-pox, 3 (2 not vaccinated); hooping-cough, 10; diarrhœa, 2; fever, 4; croup, 2; diseases of heart, 4; diseases of brain, including 7 from apoplexy, 16; convulsions, 15; diseases of liver, 3; dropsy, 2; debility and old age, 24.

"April 25. The number of deaths reported during the week was 285, being 18 more than the corrected average. Scarlatina caused 3 deaths; measles, 1; small-pox, 4; hooping-cough, 8; fever, 7; croup, 2; diarrhœa, 7; diseases of lungs, 68; diseases of heart, 10; diseases of brain, 12; convulsions, 30; paralysis, 2; dropsy, 6; excessive drinking, 1; old age, 36.

"May 2. 194 deaths were registered, being 28 less than average. Scarlatina caused 6 deaths; measles, 1; small-pox, 3; hooping-cough, 6; fever, 5; croup, 3; diarrhœa, 4; diseases of lungs, 72; diseases of heart, 3; diseases of brain, 19; convulsions, 15; diseases of the liver, 4; dropsy, 2; wasting and decay of nature, 21; old age, 3.

"May 9. There were 241 deaths registered, being 20 more than the corrected average. Scarlatina caused 11 deaths; small-pox, 4; (3 not vaccinated) hooping-cough, 14; diarrhœa, 6; typhus, 4; croup, 6; syphilis, 2; gout, 1; delirium tremens, 1; diseases of lungs, 80.

"May 16. There were 236 deaths registered during the week, being 7 more than the average. Scarlatina caused 9 deaths; measles, 1; small-pox, 3; chicken-pox, 1; hooping-cough, 12; typhus, 6; diarrhœa, 5; syphilis, 3; diabetes, 1 (a boy aged 5); delirium tremens, 1.

"May 23. The number of deaths registered during the week was 192, being about 30 less than the average. Scarlatina caused 11 deaths; small-pox, 1; hooping-cough, 16; typhus, 6; diarrhœa, 5; syphilis, 1; diseases of lungs, 54.

"May 30. There were 206 deaths registered, being 14 less than the average. Scarlatina caused 7 deaths; small-pox, 2; measles, 1; typhus fever, 13; diarrhœa, 3; diseases of lungs, 66."

*Wigan.*—Mr. Hussey writes: "Hooping-cough has prevailed at Ashton, five miles from here; the particulars I have not received. Small-pox is very rife at St. Helen's, twelve miles from Liverpool, and fatal in many cases. I had lately, in this village, a severe case of choleraic diarrhœa, with cramps and prostration, which recovered; and a remarkable case of lead palsy, from drinking water from a lead cistern. Remittent fever of children proved fatal in the majority of cases, owing to neglect in the treatment of head and chest symptoms. I cannot hear of any epidemics in cattle in this quarter. Crops in general look full and healthy. Potatoes are failing in many instances in which *sets* have been planted or sown, but not so where the seed has been *set whole.* I never saw paupers in my district so free from disease as they are at present, and, as they have been, for three months past. We have had a dreadful case of gangrenous stomatitis here within the last three months, in a locality badly ventilated and badly drained. The subject of it is a married female, aged about twenty-five years: she is still very ill, and has been so for above two months. Almost all her teeth have come out. The wind, during the quarter, was generally E., S.E., and N.W."

*Bolton-le-Moors.*—Mr. Pendlebury says: "No particular epidemic was noted during the past quarter. Cases of small-pox were far more numerous, but were confined chiefly to one or two districts, in which there was an evident neglect of vaccination. The majority were mild in type, and yielded readily to treatment. During May, diarrhœa was rather prevalent, and in many cases of a dysenteric character; error in diet was chiefly the exciting cause. Hooping-cough prevailed extensively, and from its having been, in very many cases, complicated with measles, bronchitis, and even pneumonia, increased the rate of mortality in children. A few cases, from the severity of the paroxysms, had marked signs of collapse of the lung, and congestion of the brain causing convulsions and death. I had a severe case of shingles in a female eight months advanced in pregnancy, which got well rapidly from quinine and iron, with occasional laxatives. Cases of favus of the scalp in children, and herpetic eruptions of the face in adults, were very numerous. The wind having held to the east, vegetation has been very late, but with a welcome change, and genial showers, there is a very promising appearance of healthy and abundant fruits and crops."

*Bramley.*—Mr. Radcliffe writes: "The population of Bramley, according to the census of 1851, amounts to 8,949; the height of the village above the sea is 410 feet; and the principal portion of the population are engaged in the manufacture of cloth. Hooping-cough has been epidemic throughout the winter and spring; and the epizootic, commonly termed *lung disease,* is prevalent among horned cattle. Under the term lung disease, the farmers and cattle doctors of this district include two distinct forms of disease, one being the well-known epizootic pleuro-pneumonia, the other being a febrile complaint complicated with asthenic pneumonia. In the former affection, after death, the pleura are found in an abnormal state, and the lungs are infiltrated with plastic exudation, which is often found in enormous quantity; in the latter affection the pleura rarely or never shows signs of morbid action, and the lungs are darkly congested, much softened, and the lung-tissue is often broken down and pulpy. An increased prevalence, and a greater degree of virulence of the so-called lung disease in this district during the last three or four years appears to have been due rather to a larger development of the latter affection than of pleuro-pneumonia, although cases of this disease are of frequent occurrence, and both forms of disease may exist at one and the same time among the cattle of a farm-stead. The febrile affection—*malignant catarrhal fever,* if I may so term it—like pleuro-pneumonia, is believed to be highly contagious; but this is doubtful, although it is certainly very infectious, and, like other epizootics, it haunts over-crowded, ill ventilated, and filthy sheds, and commits its greatest ravages in such places. Moreover, when it has effected a lodgment, it is most difficult to be removed; and in several farms in this vicinity it has become necessary to disuse the cattle sheds in order thoroughly to disinfect them."

*York.*—Mr. Procter says: "The latter part of the quarter was more unhealthy than the former part, and much indisposition prevailed. During April and May small-pox prevailed to a considerable extent. Carbuncular affections, some of them of considerable severity, were very prevalent."

[In the last line but one of Mr. Procter's last Report (p. 102), for "*cramp,*" read "*croup.*"]

*West Auckland.*—Mr. Todd states that during the quarter this place and the neighbourhood were unusually free from epidemic diseases. A boy, aged ten years, had scarlet fever, and a month after recovering from this he was attacked by small-pox. He had been vaccinated, and had the disease in a modified form. Scarlet fever was prevailing in the place; but Mr. Todd could not make out that the patient had ever been exposed to the contagion of small-pox.

*Rothbury.*—Mr. Summers says that the only case of erysipelas resulted from a scald in a woman of a cachectic habit. One case of puerperal fever resulted from extreme incaution on the part of the patient, after delivery of twins by turning, in a case of placenta prævia. The cases of typhus, measles, and scarlatina, especially of the two last, were mild. There was throughout the quarter a great prevalence of bilious affections, and of bronchitis, the two being frequently combined.

*Lerwick.*—Dr. Spence writes: "The first three weeks of March were cold, wet, and stormy, with the wind chiefly from the S.W., during the last week the wind prevailed from the S.E. and the weather was finer. During April S.E. winds prevailed, and the weather was showery, especially in the first part of the month. The month of May was fine though rather cold: the prevailing winds were from the S.W. and S.E. A case of small-pox was imported from Liverpool in April, but the disease did not spread. The fever that occurred was contagious, but at the same time appeared to arise from common causes, and almost all the cases originated in localities of which the sanitary state was much neglected: twenty cases came under my notice: there were three deaths, all in persons who were not removed from their houses, and who, therefore, continued exposed to the predisposing causes. The number of deaths from all causes during the quarter was 16: the average age was forty years; 4 were above seventy, and 5 below five years. The causes where I have been able to ascertain them were: gastric fever, 3; phthisis, 2; influenza, 1; erysipelas, 1; hydrocephalus, 1; convulsions, 1; debility from birth, 1; ileus, 1; and delirium tremens, 1."

---

During this quarter, the Association of Medical Officers of Health, have brought out weekly tables, showing the progress of disease in their various districts. These reports are kindred with those we have now been supplying for more than two years in this section of the *Sanitary Review*; and, in our next number, we shall embody the main facts of the metropolitan returns with ours.

One passage in the remarks on these returns, for the week ending May 23rd, is of great value.

"In last week's return it was stated that the amount of water flowing down the rivers which supply London appears to be rapidly diminishing, as evidenced by observations on the river Lea. On the 23rd inst., when the water was again measured on the weir at Stonebridge Lock, a further diminution in its depths of nearly an inch was found to have taken place in the short space of a week, and this notwithstanding the fall of ·41 in. of rain which had occurred during the same period.

"This interesting fact, first pointed out by Mr. Pittard, the officer of health for St. George-in-the-East, has doubtless a much more important bearing upon the public health than would at first sight appear. It seems highly probable that if similar observations were regularly instituted upon the amount of water flowing down the Thames, a corresponding diminution would be found to have occurred in that river also. If this supposition should prove correct, and assuming the amount of solid impurity poured into these rivers *above* the points from which the water for the supply of London is drawn by the several water companies to be *constant*, it follows that when the quantity of water has been thus reduced (as during the last six weeks has actually been the case) no less than *two-thirds* its previous amount, a given quantity of drinking-water must contain *one-third* more of solid impurity than it did six weeks ago. May not this circumstance have some definite relation to, and in some measure at least afford an explanation of, the amount of diarrhœa which so constantly prevails in this metropolis during the summer months ?"

The sheet of the Metropolitan Officers of Health contains also a return of the meteorology of London. There are here some inaccuracies. It would be well if this department (should the scheme progress) were put under the entire direction of Mr. Glaisher.

